# Viral suppression by residency status among men living with HIV in the context of the expanded ART policy in Shanghai, China

**DOI:** 10.1371/journal.pgph.0005942

**Published:** 2026-02-12

**Authors:** Joshua B. Mendelsohn, Yinzhong Shen, Min Xi, Hua Cheng, Sandra Bullock, Xueyun Wu, Ann N. Burchell, Sharmistha Mishra, Darrell H. S. Tan, Mona Loutfy, Veronika Moravan, Liviana Calzavara

**Affiliations:** 1 College of Health Professions, Pace University, New York, New York, United States of America; 2 Department of Infection and Immunity, Shanghai Public Health Clinical Center, Shanghai, China; 3 Dalla Lana School of Public Health, University of Toronto, Toronto, Ontario, Canada; 4 ICES, Toronto, Ontario, Canada; 5 Shanghai Municipal Centre for Disease Control and Prevention, Shanghai, China; 6 MAP Centre for Urban Health Solutions, Unity Health Toronto, Toronto, Ontario, Canada; 7 Department of Family and Community Medicine, Temerty Faculty of Medicine, University of Toronto, Toronto, Ontario, Canada; 8 Institute of Health Policy, Management, and Evaluation, University of Toronto, Toronto, Ontario, Canada; 9 Division of Infectious Diseases, St Michael’s Hospital, Unity Health Toronto, Toronto, Ontario, Canada; 10 Department of Medicine, Faculty of Medicine, University of Toronto, Toronto, Ontario, Canada; 11 Institute of Medical Science, University of Toronto, Toronto, Ontario, Canada; 12 Women’s College Hospital, Toronto, Ontario, Canada; 13 VM Stats, Toronto, Ontario, Canada; University of California Irvine, UNITED STATES OF AMERICA

## Abstract

Access to China’s national free antiretroviral therapy (ART) program was historically restricted to the home province or province of birth (“Hukou”). During the COVID-19 pandemic, Shanghai facilitated access to free ART and supportive services for all Shanghai residents regardless of residency status. Our aim was to study differences in viral suppression according to residency status in Shanghai, China. We conducted a cross-sectional survey with a nine-month prospective follow-up for viral load testing. Viral suppression was defined as a viral load of <40 copies/mL. Data were analyzed using descriptive statistics and Firth logistic regression. We recruited 151 adult men newly diagnosed with HIV from a downtown satellite clinic of the major HIV reference hospital in Shanghai. Among recruited participants, 23.8% were Shanghai Hukou residents, 23.2% were external permanent residents, 16.6% were temporary migrants, and 36.4% had no Shanghai residency status. Almost all participants (98.7%) initiated ART; 72.8% had an undetectable viral load by nine-months following ART initiation. The median time from HIV diagnosis to ART initiation was shorter for Shanghai Hukou residents (9.0 days) compared to other groups (15.0-19.0 days, p = 0.023). Shanghai Hukou residents (aOR: 0.24; 95% CI: 0.05, 1.00; p = 0.05), temporary migrants (aOR:0.20; 95% CI: 0.04, 0.92; p = 0.038), and men with no Shanghai residence identification (aOR:0.16; 95% CI: 0.03, 0.61; p = 0.006) were less likely to attain viral suppression when compared with external permanent residents, after adjusting for sociodemographic, sexual behaviour, and clinical covariates. Overall, differences in viral suppression between residency groups persisted despite the new policy of free ART regardless of residency status. These disparities highlight the need to better understand the underlying dynamics of viral suppression with attention to residency status. Future work should focus on developing complementary interventions and expanding supportive services to facilitate rapid viral suppression among all residency groups to achieve optimal population health outcomes.

## Introduction

In 2020, there were approximately 376 million Chinese internal migrants (i.e., those who left their home province to live elsewhere in China; approximately 27% of the total Chinese population), of whom 249 million were rural-to-urban migrant workers [1]. In 2023, approximately half of the population (42%) in Shanghai, the largest city in China, was made up of migrants [2]. Fifty-five percent of internal migrants in Shanghai were male [2]. Given the scale of migration in China, the health of internal migrants has become a growing priority for national and local Chinese governments in recent years [3,4].

The number of people living with HIV in China has continued to increase since 2012, reaching 1.29 million by the end of 2023 [5]. Chinese internal migrants were found to have between a 2.63 to 6.70 times higher odds of prevalent HIV infection compared to the wider population in China [6]. Sexual transmission has been the main mode of transmission in China [7]. Mobility between regions leads to separation from long-term partners [8–11], and has been associated with sexual partnerships with an increased risk of HIV acquisition (low condom use [12,13], multiple partners [8–11], paid sex [8–11]). Male internal migrants in Shanghai were found to have lower odds of condom use at first sex and consistent condom use with their first partner compared to non-migrants [14]. Consistent ART use, resulting in an undetectable viral load, can improve the quality of life and life expectancy of people living with HIV [15]. Given higher HIV prevalence and incidence among Chinese migrants compared to non-migrants, consistent ART use leading to viral suppression is needed to reduce HIV transmission. Migrants have been a priority for HIV prevention in China, but there remains a significant gap in the development and implementation of intervention strategies that are specifically tailored to address the unique challenges and needs of migrant populations [16,17].

Prior to the COVID-19 pandemic in China, provision of HIV testing, treatment, and care through publicly funded and government-designated clinics and hospitals depended on residency status [18]. There are four types of residency status in Shanghai: registered permanent residents, external permanent residents, temporary migrants, and those with no Shanghai residence identification [19]. Registered permanent residents (referred to as “Shanghai Hukou” residents) were either born in Shanghai with Shanghai household registration status (i.e., “Hukou”) or were born outside of Shanghai and obtained a Shanghai Hukou after immigrating to the city. External permanent residents were born outside of Shanghai but obtained a Shanghai residence card after immigrating to the city and are typically well educated [20]. Temporary migrants were born outside of Shanghai and immigrated to Shanghai without obtaining a Shanghai Hukou or permanent resident status; instead they obtained a temporary identification card typically valid for six months [18]. Those without Shanghai residence identification were not able to register for any formal residency status classification in Shanghai.

As part of routine care prior to the COVID-19 pandemic, Shanghai Hukou residents and external permanent migrants had free access to HIV testing, treatment, and care; those who received a confirmatory HIV diagnosis through the Shanghai Centre for Disease Control and Prevention (SCDC) were referred to the Shanghai Public Health Clinical Center (SPHCC) to receive CD4 testing (twice per year) and were offered free ART [21–23]. Once initiated on ART, they were contacted for follow-up at two-weeks, one-, two-, three-, and six-months post-ART initiation and every three months thereafter. At six-months post-ART, Shanghai Hukou and external permanent residents were eligible for their first free viral load test, and yearly thereafter. Viral load and CD4 testing were available, but access was limited if ART was not initiated. Prior to the COVID-19 pandemic, temporary migrants and those with no Shanghai residence identification could access voluntary counselling and testing in Shanghai, but were referred to their home provinces for free access to HIV care offered by the national HIV program [24]. Temporary migrants and those without any Shanghai residency status could pay out-of-pocket to receive HIV care in Shanghai, but this may have resulted in financial barriers. Prior to the COVID-19 pandemic, studies found that having a local Hukou [25,26] or residence permit [27] was associated with an increased likelihood of ART initiation and viral suppression. Rural-to-urban migrants reported that urban Hukou holders had access to better HIV treatment and services [28]. When provided with similar access to services, studies have found that migrants living with HIV demonstrated similar clinical outcomes to other local population groups, but none have been conducted in a Chinese setting [29,30].

From January 26, 2020, to December 5, 2022, due to COVID-19 pandemic-related public health restrictions, China transitioned from a policy of free ART provision for internal migrants that was restricted to their home province to an unrestricted, person-centered policy that initially provided one month of free ART at any local HIV clinic or hospital where migrants lived and worked [18,31]. If patients were unable to return to their home province for ART after one month, they could continue to receive ART locally on a temporary basis. Following the onset of the COVID-19 pandemic, national guidelines required public health units to contact people living with HIV with whom they had not regularly followed up.

The future of HIV care in China is at a critical juncture as the Chinese government contemplates the future of unrestricted free ART provision across the country. To the best of our knowledge, no Chinese studies have investigated the impact of residency status on viral suppression following these policy changes. We aimed to compare viral suppression among migrant men without residence identification in Shanghai to those with formal identification including Shanghai Hukou, external permanent status, and temporary status from 2021 to 2022. We hypothesized that unrestricted access to ART and viral load testing would result in similar levels of viral suppression among Chinese citizens despite differences in residency status.

## Materials and methods

### Study setting

We conducted a cross-sectional survey with a prospective viral load follow-up of HIV-positive men in Shanghai, China. Our study was conducted at the downtown satellite clinic operated by the SPHCC, located in the Hongkou District of Shanghai. The SPHCC is the tertiary public health hospital where most HIV-related care is delivered in Shanghai following any confirmatory HIV test result. Eligible clients, recruited from the SPHCC from May 11, 2021, to March 7, 2022, completed an interviewer administered cross-sectional survey and were followed-up by chart extraction for nine-months (until December 7, 2022).

### Participants and recruitment

Following check-in, new clients were approached sequentially by a trained interviewer in the waiting room pending interviewer availability, informed about the study, and invited to participate in an eligibility screening. Clients were eligible to participate if they: (1) were men (male sex at birth or transgender men) aged 18 years or older, (2) were current residents of Shanghai (Shanghai Hukou resident, external permanent resident, temporary migrant, or no Shanghai residence identification), (3) could speak and comprehend Mandarin enough to be able to understand the consent form and survey, and (4) were within 60 days of receiving their confirmatory HIV diagnosis in Shanghai. We excluded clients who were incapable of giving informed consent, including those who required a guardian or other third-party to make healthcare decisions.

### Data collection

All participants who provided written consent completed a baseline survey. Chart extractions were completed after nine-months of observation. Participants had the option of completing the baseline survey immediately after providing informed consent or scheduling an appointment to do so. Of 151 study participants, 147 (97.4%) completed the survey immediately following eligibility screening; remaining participants completed the survey within one week of screening. Survey responses were captured using pen and paper by a trained interviewer in a private room.

The questionnaire collected information on socio-demographics (i.e., age, marital status, education, income), sexual behaviour (i.e., ever had consensual sexual activity with men only, women only, or with men and women, concurrent sexual partnerships), and self-reported residency status. The four self-reported residency status categories included: Shanghai Hukou resident, external permanent resident, temporary migrant, and no Shanghai residence identification. Further, participants self-reported whether they received any support for their HIV diagnosis (yes/no), and the perceived number of barriers to HIV care (number of yes’s/no’s to the following: cost of care, travel times/cost, clinic hours of operation, length of appointment, difficulty taking time off work, negative attitudes from healthcare providers, and other). Participants also self-reported whether they were aware of ART, their beliefs about ART (agree/disagree with whether: ART reduces the amount of HIV virus in the body, consistent ART use will reduce the chances of transmitting HIV to sexual partners, and ART can help individuals achieve a normal life expectancy), and ART cost beliefs (agree/disagree with whether: ART is free in Shanghai for Shanghai Hukou and external permanent residents and ART is free for all Chinese citizens in their home province). These were modelled as covariates in data analyses.

Chart extraction provided information on the date of confirmatory HIV-diagnosis test, date of ART initiation, ART medications, and viral load and CD4 test dates and results. The primary outcome was viral suppression defined as <40 copies/mL within 270 days (approximately nine months) following ART initiation. Our chart extractions showed that some participants received their first viral load test prior to the routine six-months after ART initiation, some tested after six-months, and others only tested after our nine-month study follow-up period and therefore had no recorded study result. Participants who started ART earlier and who tested earlier in the follow-up period had more opportunities to be re-tested within the nine-month follow-up; those who started ART later may have been less likely to suppress within the study timeframe. Those who started ART earlier and tested later in the follow-up period may have been more likely to suppress within the study timeframe; however, if they were unsuppressed, they were less likely to have an opportunity to retest within the nine-month follow-up. To control for irregularity in viral load testing, we calculated “weeks from last viral load test to end of observation period” which estimated the number of weeks between last viral load test following ART initiation to the end of the nine-month study follow-up period (i.e., 38.6 weeks or 270 days). The number of weeks from last viral load to end of the study observation period was set to 38.6 for participants who had no viral load tests within this period.

CD4 test dates and results, measured using routine flow cytometry, were extracted from clinical charts and modelled as covariates. Plasma HIV-1 RNA viral load was measured with one of two tests: the Abbott m2000 Real Time HIV-1 Test (Abbott Molecular, Des Plaines, IL, USA; with a lower limit of detection of 40 copies/mL); and Cobas HIV-1 Test (Roche Diagnostics, Indianapolis, IN, USA; with the lower limit of detection of 20 copies/mL). All baseline survey and chart extraction data were double entered into Epidata EntryClient 4.6, (EpiData Association, Denmark, https://epidata.dk/) by study staff.

### Statistical analyses

Descriptive statistics were generated for all covariates of interest. Association between the four residence groups and covariates were tested using chi-square or Fisher’s exact test for categorical, Kruskal-Wallis test for ordinal, or ANOVA for continuous variables with Scheffe post-hoc tests [32]. Firth logistic regression, the standard analytical method for handling binary outcomes in small datasets [33], was used to obtain crude and adjusted odds ratios for the association between Shanghai residency status and viral suppression (undetectable viral load conservatively dichotomized as: < 40 copies/mL versus detectable/unknown) within 270 days following ART initiation, adjusting for covariates of interest. Sixteen participants who did not undergo viral load testing over the nine-month study period were conservatively classified as having a detectable/unknown viral load in our primary analysis. We used backward elimination to specify the model by removing the covariate with the highest penalized likelihood ratio test p-value at each step, until only covariates with p < 0.10, and pre-determined control variables remained. In sensitivity analyses, we excluded 16 participants with an unknown viral load from our regression model. p-values were two-sided and p < 0.05 was used to determine statistical significance, except for p < 0.10 in retaining covariates during backwards elimination. Analyses were conducted using R software version 4.3.2 (R Foundation for Statistical Computing, Vienna, Austria) [34].

### Ethical approval

This study received ethics approval from the University of Toronto Research Ethics Board (REB; Protocol #35006) and the SPHCC Ethics Committee (Protocol #2019-S033-02).

## Results

### Participant characteristics

We approached 409 clients at the downtown satellite of the SPHCC; 55 (13.4%) declined to participate after hearing a brief project description, the remaining 354 clients (86.6%) were screened for eligibility. After screening, 163/354 (46.0%) met eligibility criteria, and 151 men (92.6%) enrolled in the study; our analysis is focused on these 151 participants.

At baseline, 35 (23.2%) participants were external permanent residents, 36 (23.8%) were Shanghai Hukou residents, 25 (16.6%) were temporary migrants , and 55 (36.4%) had no Shanghai residence identification (Table 1). The mean baseline CD4 count was 311 cells/µL (standard deviation [SD]: 168). The mean age of participants was 32.1 (SD: 11.3) but differed between residence groups (ANOVA p < 0.001). External permanent migrants had a mean age of 30.4 (SD: 6.6). This was similar to the mean age of temporary migrants 29.2 (SD: 7.6) and men without Shanghai residency status 28.3 (SD: 8.5), but Shanghai Hukou residents tended to be older (mean: 41.8; SD: 15.2; Scheffe p < 0.05). There was some evidence for a difference in marital status between groups (Fisher’s exact test p = 0.055); the proportion of participants who were single/never married was lowest among Shanghai Hukou residents (55.6%), followed by external permanent residents (68.6%), temporary migrants (76.0%), and those with no Shanghai residence identification (85.5%). There were differences in the gender of consensual lifetime sex partners (Fisher’s exact test p = 0.022); 54.3% of external permanent residents had sex with men only compared to 47.2% of Shanghai Hukou residents, 68.0% among temporary migrants, and 69.1% among those with no Shanghai residence identification. Among those with no Shanghai residence identification, 1.8% had sex with women only; this proportion ranged from 8.6 to 22.2% in the other three groups. A lower proportion of external permanent residents were involved in concurrent relationships compared to Shanghai Hukou residents, temporary migrants, and those without Shanghai residence identification (42.9% versus 44.4 – 72.7%, Fisher’s exact test p = 0.012).

**Table 1:** Participant characteristics by Shanghai residency status (N = 151).

	Total N = 151 n (%)	Shanghai Residency Status				p-value
		External permanent n = 35 n (%)	Shanghai Hukou n = 36 n (%)	Temporary n = 25 n (%)	No Shanghai residence identification n = 55 n (%)	
Age at Interview						
Mean (SD)	32.1 (11.3)	30.4 (6.6)	41.8 (15.2)	29.2 (7.6)	28.3 (8.5)	**F = 14.74,** **df = 3, 147** **p < 0.001** ^**a**^
Marital status						
Single/Never married	110 (72.8)	24 (68.6)	20 (55.6)	19 (76.0)	47 (85.5)	0.055^**b**^
Married	29 (19.2)	7 (20.0)	11 (30.6)	4 (16.0)	7 (12.7)	
Former married and other	12 (7.9)	4 (11.4)	5 (13.9)	2 (8.0)	1 (1.8)	
Education						
≤ High school	33 (21.9)	7 (20.0)	8 (22.2)	5 (20.0)	13 (23.6)	0.180^b^
Vocational school/college	92 (60.9)	20 (57.1)	21 (58.3)	20 (80.0)	31 (56.4)	
Professional undergraduate/graduate degree	26 (17.2)	8 (22.9)	7 (19.4)	0 (0)	11 (20.0)	
Had consensual sex with						
Men only	91 (60.3)	19 (54.3)	17 (47.2)	17 (68.0)	38 (69.1)	**0.022** ^**b**^
Women only	16 (10.6)	3 (8.6)	8 (22.2)	4 (16.0)	1 (1.8)	
Both men and women	44 (29.1)	13 (37.1)	11 (30.6)	4 (16.0)	16 (29.1)	
CD4 measure at baseline						
N	150	35	35	25	55	
Mean (SD)	311 (168)	344 (190)	283 (155)	296 (182)	315 (156)	F = 0.85 df = 3, 146 p = 0.466^a^
Income (in 1000’s)^c^						
N	151	35	36	25	55	
Median (IQR)	10.0 (6.5, 15.0)	12.0 (9.0, 20.0)	10.0 (4.8, 18.0)	10.0 (6.5, 15.0)	10.0 (6.0, 13.8)	KW = 5.62, df = 3 p = 0.132^d^
Concurrent partnerships						
Yes	84 (55.6)	15 (42.9)	16 (44.4)	13 (52.0)	40 (72.7)	**0.012** ^**b**^
No/No sex	67 (44.4)	20 (57.1)	20 (55.6)	12 (48.0)	15 (27.3)	
Any source of HIV support						
Yes, has support	120 (79.5)	24 (68.6)	31 (86.1)	22 (88.0)	43 (78.2)	0.223^b^
No support/Not stated	31 (20.5)	11 (31.4)	5 (13.9)	3 (12.0)	12 (21.8)	
Barriers to HIV-related care						
None	81 (53.6)	21 (60.0)	17 (47.2)	14 (56.0)	29 (52.7)	0.766^b^
1 barrier	32 (21.2)	5 (14.3)	7 (19.4)	6 (24.0)	14 (25.5)	
2 or more barriers	38 (25.2)	9 (25.7)	12 (33.3)	5 (20.0)	12 (21.8)	
Heard about ART for HIV - Yes	97 (64.2)	22 (62.9)	24 (66.7)	16 (64.0)	35 (63.6)	0.990^b^
ART beliefs: medical^e^						
None	56 (37.1)	13 (37.1)	13 (36.1)	9 (36.0)	21 (38.2)	0.996^b^
1 or 2	34 (22.5)	9 (25.7)	7 (19.4)	6 (24.0)	12 (21.8)	
All 3	61 (40.4)	13 (37.1)	16 (44.4)	10 (40.0)	22 (40.0)	
ART beliefs: cost^f^						
None	57 (37.7)	13 (37.1)	12 (33.3)	10 (40.0)	22 (40.0)	0.457^b^
1	36 (23.8)	10 (28.6)	5 (13.9)	5 (20.0)	16 (29.1)	
Both	58 (38.4)	12 (34.3)	19 (52.8)	10 (40.0)	17 (30.9)	
Weeks from last viral load test to end of observation period^g^						
Mean (SD)	18.9 (8.9)	19.6 (8.4)	21.2 (9.3)	17.9 (8.5)	17.4 (8.9)	F = 1.51 df = 3, 147 p = 0.215^a^
Time from confirmatory HIV test to ART initiation (days)^h^						
Median (IQR)	15.0 (9.0, 27.0)	15.0 (9.5, 29.5)	9.0 (4.8, 20.2)	19.0 (12.0, 30.0)	16.0 (10.0, 24.0)	KW = 9.54 df = 3 p = 0.023^d^

^a^ Analysis of variance test (ANOVA).

^b^ Fisher’s exact test.

^c^ N = 3 missing were imputed with median.

^d^ Kruskal-Wallis test.

^e^ Participants were asked if they agreed or disagreed with each of the following statements: 1) ART reduces the amount of HIV virus, 2) consistent ART use will reduce the chances of transmitting HIV to sexual partners, and 3) ART can help individuals achieve a normal life expectancy.

^f^ Participants were asked if they agreed or disagreed with each of the following statements: 1) ART is free in Shanghai for Shanghai Hukou and external permanent residents and 2) ART is free for all Chinese citizens in their home province.

^g^ Set to 38.6 weeks (270 days) if no viral load tests were in follow-up range (n = 16).

^h^ Set to 270 days if participant never started ART (n = 2).

Abbreviations: N = number of participants; SD = standard deviation; IQR = interquartile range; F = F-statistic; df = degrees of freedom; KW = Kruskal-Wallis statistic; p = p-value.

### ART use and subsequent viral load testing

Of the 151 men, 149 (98.7%) initiated ART and 132 (87.4%) initiated ART within one week of survey completion. The median time from HIV diagnosis to ART initiation was 15.0 days (IQR: 9.0, 27.0), with a median of 9.0 days (IQR: 4.8, 20.2) for Shanghai Hukou residents versus 15.0 days (IQR: 9.5, 29.5) for external permanent migrants, 19.0 days (IQR: 12.0, 30.0) for temporary migrants, and 16.0 days (IQR: 10.0, 24.0) for men without Shanghai residence identification (Kruskal-Wallis test p = 0.023). The most common ART regimen used by participants was efavirenz/lamivudine/tenofovir disoproxil fumarate (EFV/3TC/TDF; n = 105; 69.5%), followed by elvitegravir/cobicistat/emtricitabine/tenofovir alafenamide (EVGc-FTC-TAF; n = 14; 9.3%), and bictegravir/emtricitabine/tenofovir (BIC-FTC-TAF; n = 10; 6.6%).

**Fig 1:**
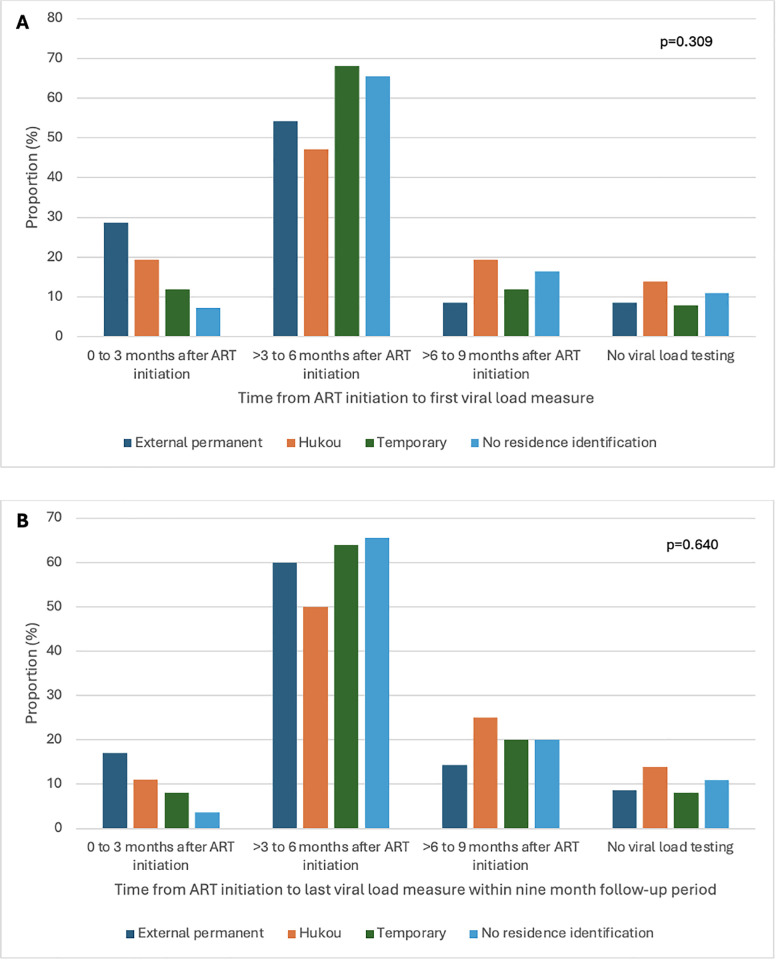
(A) Proportion of each residence group having their first viral load measures within three-month intervals within the nine-month follow-up period (N = 151). Proportion of each residence group having their last viral load measures within three-month intervals within the nine-month follow-up period (N = 151).

Most participants (89.4%; 135/151) received a viral load test within nine months following ART initiation; but 10.6% (16/151) did not receive a viral load test during this period (Fig 1A and 1B). Baseline characteristics did not differ between participants who did versus those who did not receive a viral load test within nine months following ART initiation when comparing Shanghai residence (p = 0.889), age (p = 0.279), marital status (p = 0.440), education (p = 0.117), income (p = 0.160), consensual sex partner gender (p = 0.528), concurrent sexual partners (p = 0.791), concurrent male/female sex partners (p = 0.753), baseline CD4 (p = 0.879), any source of HIV support (p = 0.527), barriers to HIV-related care (p = 0.196), heard about ART for HIV (p = 0.271), ART beliefs – medical (p = 0.144), or ART beliefs – cost (p = 0.204). The mean number of weeks from last viral load test to end of the observation period was 18.9 weeks (SD: 8.9) for all participants and 19.6 (SD: 8.4) for external permanent residents, 21.2 weeks (SD: 9.3) for Shanghai Hukou residents, 17.9 weeks (SD: 8.5) for temporary migrants and 17.4 weeks (SD: 8.9) for those with no Shanghai residence identification (ANOVA p = 0.215; Table 1).

### Factors associated with viral suppression

**Fig 2:**
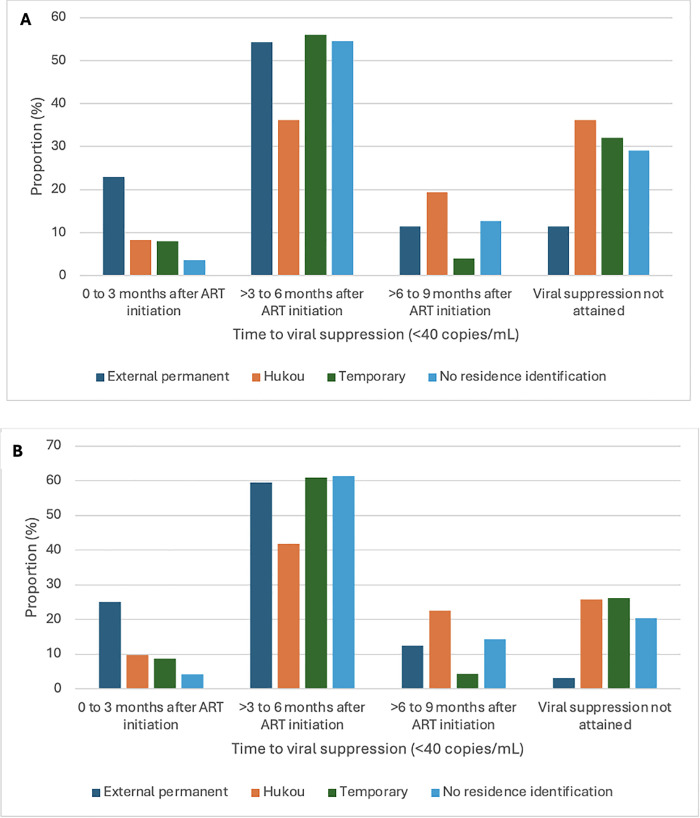
(A). Proportion of each residence group reaching viral suppression (<40 copies/mL) within three-month intervals within the nine-month follow-up period for all participants (N = 151). Fisher’s exact p-value: 0.044. (B) Proportion of each residence group reaching viral suppression (<40 copies/mL) within three-month intervals within the nine-month follow-up period for the 135 participants with a viral load test result. Fisher’s exact p-value: 0.046.

Within nine months of ART initiation, almost three-quarters (72.8%; 110/151) of participants were virally suppressed (<40 copies/mL; Fig 2A and 2B). The proportion of participants who attained viral suppression varied by residency status with the largest proportion suppressed among external permanent residents (85.7%). Almost two thirds (63.9%) of Shanghai Hukou residents were virally suppressed, 68.0% temporary migrants, and 70.9% men without Shanghai residence identification (Table 2). In the crude logistic regression model, there was weak evidence for association of Shanghai residency status with viral suppression. After adjusting for socio-demographic, sexual behaviour, and clinical variables, there was strong evidence for this association (p = 0.044). Compared to external permanent residents, Shanghai Hukou residents (aOR: 0.24; 95% CI: 0.05, 1.00; p = 0.050), temporary migrants (aOR:0.20; 95% CI: 0.04, 0.92; p = 0.038), and men with no Shanghai residence identification (aOR: 0.16; 95% CI: 0.03, 0.61; p = 0.006) achieved lower odds of viral suppression; Table 2). With 16 participants without a viral load measure removed from the analyses, 81.5% (110/135) men were virally suppressed during the observation period. Adjusted odds ratios remained similar in these sensitivity analyses, but the Shanghai residency status variable was no longer statistically significant (p = 0.186; Table 3).

**Table 2:** Odds of receiving an undetectable viral load (<40 copies/mL) by 9-months post ART initiation, by Shanghai residency status^a^.

	n	Achieved undetectable viral load by 9 months after ART initiation N (%)	Crude odds ratios (N = 151)			Adjusted odds ratios (N = 150)		
			OR (95% CI)	Z-test p-value^b^	PLR test p-value^c^	aOR (95% CI)	Z-test p-value^b^	PLR test p-value^c^
**Shanghai residence**					0.084			**0.044**
External permanent	35	31 (88.6)	1			1		
Hukou	36	23 (63.9)	**0.25 (0.07, 0.77)**	**0.016**		**0.24 (0.05, 1.00)**	**0.050**	
Temporary	25	17 (68.0)	0.29 (0.08, 1.03)	0.055		**0.20 (0.04, 0.92)**	**0.038**	
No residence identification	55	39 (70.9)	0.34 (0.10, 1.00)	0.051		**0.16 (0.03, 0.61)**	**0.006**	
**Age at interview** *(centered, scaled by 5 years)*	151	–	0.97 (0.83, 1.13)	0.672	0.672	1.06 (0.78, 1.46)	0.690	0.690
**Marital status**					0.397			0.108
Single/Never married	110	80 (72.7)	1			1		
Married	29	23 (79.3)	1.37 (0.55, 3.85)	0.515		1.15 (0.21, 6.99)	0.873	
Former married	12	7 (58.3)	0.52 (0.16, 1.76)	0.280		0.17 (0.02, 1.16)	0.071	
**Education**					0.838			0.946
≤ High school	33	23 (69.7)	1			1		
Vocational school/college	92	67 (72.8)	1.18 (0.49, 2.75)	0.703		0.90 (0.23, 3.25)	0.874	
Professional undergraduate/graduate degree	26	20 (76.9)	1.41 (0.46, 4.61)	0.554		0.77 (0.15, 3.75)	0.744	
**Had consensual sex with**					0.111			0.187
Men only	91	63 (69.2)	1			1		
Women only	16	10 (62.5)	0.73 (0.25, 2.22)	0.561		0.67 (0.13, 3.80)	0.634	
Both men and women	44	37 (84.1)	2.24 (0.95, 5.86)	0.066		2.39 (0.68, 10.01)	0.180	
**CD4 measure at baseline** *(centered, scaled by 100)*	150	–	**1.26 (1.00, 1.62)**	**0.047**	**0.047**	**1.41 (1.07, 1.95)**	**0.0131**	**0.0131**
**Weeks from last viral load test to end of observation period** ^**d**^	151	–	**0.90 (0.85, 0.94)**	**<0.0001**	**<0.0001**	**0.89 (0.84, 0.93)**	**<0.0001**	**<0.0001**
**Income** *(centered, in 1000’s)^e^*	151	–	1.00 (0.99, 1.01)	0.947	0.947			
**Concurrent partnerships**					0.505			
Yes	84	63 (75.0)	1					
No/No sex	67	47 (70.1)	0.78 (0.38, 1.60)	0.505				
**Concurrent male-female partnerships**					0.473			
Yes	12	10 (83.3)	1					
No/No sex	139	100 (71.9)	0.61 (0.11, 2.21)	0.473				
**Any source of HIV support**					0.298			
Yes, has support	120	85 (70.8)	1					
No support/Not stated	31	25 (80.6)	1.63 (0.66, 4.52)	0.298				
**Barriers to HIV-related care**					0.345			
None	81	61 (75.3)	1					
1 barrier	32	20 (62.5)	0.55 (0.23, 1.31)	0.174				
2 or more	38	29 (76.3)	1.04 (0.43, 2.59)	0.939				
**Time since first HIV test** *(months)*	151	–	1.01 (1.00, 1.02)	0.122	0.122			
**Time from confirmatory HIV test to ART initiation** *(days)*^f^	151	–	0.99 (0.98, 1.00)	0.085	0.085			
**Heard about ART for HIV**					0.203			
Yes	97	74 (76.3)	1					
No	54	36 (66.7)	0.62 (0.30, 1.29)	0.203				
**ART beliefs: medical** ^**g**^					0.395			
None	56	38 (67.9)	1					
1 or 2	34	24 (70.6)	1.12 (0.46, 2.85)	0.805				
All 3	61	48 (78.7)	1.73 (0.77, 3.98)	0.189				
**ART beliefs: cost** ^**h**^								
None	57	39 (68.4)	1		0.261			
1	36	30 (83.3)	2.20 (0.83, 6.44)	0.115				
Both	58	41 (70.7)	1.11 (0.51, 2.45)	0.793				

^a^ Includes all participants, those with no viral load measure were conservatively imputed as detectable.

^b^ Z-test for null hypothesis that the coefficient (parameter) is zero.

^c^ Penalized likelihood ratio test.

^d^ Set to 38.6 weeks (270 days) if no viral load tests in follow-up range.

^e^ N = 3 missing were imputed with median.

^f^ Set to 270 days if participant never started ART (n = 2).

^g^ Participants were asked if they agreed or disagreed with each of the following statements: 1) ART reduces the amount of HIV virus, 2) consistent ART use will reduce the chances of transmitting HIV to sexual partners, and 3) ART can help individuals achieve a normal life expectancy.

^h^ Participants were asked if they agreed or disagreed with each of the following statements: 1) ART is free in Shanghai for Shanghai Hukou and external permanent residents and 2) ART is free for all Chinese citizens in their home province.

**Table 3:** Odds of receiving an undetectable viral load (<40 copies/mL) by 9-months post ART initiation, by Shanghai residency status, excluding participants without a viral load test result (N = 135).

	n	Achieved undetectable viral load by 9 months after ART initiation N (%)	Crude odds ratios (N = 135)			Adjusted odds ratios (N = 135)		
			OR (95% CI)	Z-test p-value^a^	PLR test p-value^b^	aOR (95% CI)	Z-test p-value^a^	PLR test p-value^b^
**Shanghai residence**					**0.042**			0.186
External permanent	32	31 (96.9)	1			1		
Hukou	31	23 (74.2)	**0.13 (0.01, 0.65)**	**0.010**		**0.19 (0.02, 0.98)**	**0.047**	
Temporary	23	17 (73.9)	**0.13 (0.01, 0.68)**	**0.014**		0.23 (0.02, 1.37)	0.110	
No residence identification	49	39 (79.6)	**0.18 (0.02, 0.83)**	**0.026**		0.22 (0.02, 1.09)	0.066	
**Age at interview** *(centered, scaled by 5 years)*	135	–	1.06 (0.87, 1.34)	0.594	0.594	1.09 (0.78, 1.57)	0.612	0.612
**Marital status**					0.237			0.120
Single/Never married	100	80 (80.0)	1			1		
Married	25	23 (92.0)	2.39 (0.69, 12.54)	0.181		1.57 (0.24, 13.53)	0.645	
Former married	10	7 (70.0)	0.55 (0.15, 2.41)	0.397		0.18 (0.02, 1.29)	0.087	
**Education**					0.590			0.875
≤ High school	26	23 (88.5)	1			1		
Vocational school/college	85	67 (78.8)	0.54 (0.13, 1.70)	0.311		0.77 (0.14, 3.38)	0.742	
Professional undergraduate/graduate degree	24	20 (83.3)	0.68 (0.14, 3.12)	0.615		0.63 (0.10, 3.64)	0.606	
**Had consensual sex with**					0.089			0.324
Men only	82	63 (76.8)	1			1		
Women only	13	10 (76.9)	0.92 (0.27, 3.94)	0.903		0.75 (0.12, 5.56)	0.765	
Both men and women	40	37 (92.5)	**3.29 (1.09, 13.07)**	**0.033**		2.28 (0.63, 10.52)	0.217	
**CD4 measure at baseline** *(centered, scaled by 100)*	135	–	**1.48 (1.09, 2.10)**	**0.010**	**0.010**	**1.50 (1.08, 2.21)**	**0.015**	**0.015**
**Weeks from last viral load test to end of observation period**	135	–	0.99 (0.93, 1.07)	0.852	0.852	1.00 (0.92, 1.08)	0.925	0.925
**Income** *(centered, in 1000’s)*^c^	135	–	1.00 (0.99, 1.05)	0.684	0.684			
**Concurrent partnerships**					0.626			
Yes	76	63 (82.9)	1					
No/No sex	59	47 (79.7)	0.81 (0.34, 1.92)	0.626				
**Concurrent male-female partnerships**					0.530			
Yes	11	10 (90.9)	1					
No/No sex	124	100 (80.6)	0.59 (0.06, 2.69)	0.530				
**Any source of HIV support**					0.521			
Yes, has support	106	85 (80.2)	1					
No support/Not stated	29	25 (86.2)	1.43 (0.51, 4.86)	0.521				
**Barriers to HIV-related care**					0.712			
None	73	61 (83.6)	1					
1 barrier	26	20 (76.9)	0.64 (0.22, 1.97)	0.423				
2 or more barriers	36	29 (80.6)	0.80 (0.30, 2.27)	0.665				
**Time since first HIV test** *(months)*	135	–	1.01 (1.00, 1.02)	0.243	0.243			
**Time from confirmatory HIV test to ART initiation** *(days)*^d^	135	–	1.00 (0.98, 1.02)	0.966	0.966			
**Heard about ART for HIV**					0.473			
Yes	89	74 (83.1)	1					
No	46	36 (78.3)	0.72 (0.30, 1.78)	0.473				
**ART beliefs: medical** ^**e**^					0.885			
None	48	38 (79.2)	1					
1 or 2	29	24 (82.8)	1.22 (0.40, 4.09)	0.739				
All 3	58	48 (82.8)	1.26 (0.48, 3.31)	0.636				
**ART beliefs: cost** ^**f**^					0.774			
None	49	39 (79.6)	1					
1	35	30 (85.7)	1.47 (0.49, 4.90)	0.498				
Both	51	41 (80.4)	1.05 (0.40, 2.77)	0.920				

^a^ Z-test for null hypothesis that the coefficient (parameter) is zero.

^b^ Penalized likelihood ratio test.

^c^ N = 3 missing were imputed with median.

^d^ Set to 270 days if participant never started ART (n = 2).

^e^ Participants were asked if they agreed or disagreed with each of the following statements: 1) ART reduces the amount of HIV virus, 2) consistent ART use will reduce the chances of transmitting HIV to sexual partners, and 3) ART can help individuals achieve a normal life expectancy.

^f^Participants were asked if they agreed or disagreed with each of the following statements: 1) ART is free in Shanghai for Shanghai Hukou and external permanent residents and 2) ART is free for all Chinese citizens in their home province.

In adjusted analyses, there was strong evidence for associations of viral suppression with baseline CD4 count and weeks from last viral load test to end of observation period (Table 2). For each 100 cells/µL increase in baseline CD4 count (aOR=1.41; 95% CI: 1.07, 1.95, p = 0.013) odds of viral suppression increased. For each additional week from last viral load test to end of observation period (aOR=0.89; 95% CI: 0.84, 0.93, p < 0.0001), odds of viral suppression decreased. In sensitivity analyses, only baseline CD4 count remained statistically significant (aOR=1.50; 95% CI: 1.08, 2.21; p = 0.015; Table 3).

## Discussion

Our study found high proportions of participants initiating ART and moderate levels of viral suppression within nine-months after HIV diagnosis among men living in Shanghai, China. Despite high ART initiation, we identified differences in viral suppression by Shanghai residency status, suggesting a need for ongoing surveillance to further understand this inequality. We also found that higher baseline CD4 counts and fewer weeks from last viral load test to the end of the observation period were associated with viral suppression.

The proportion of participants who initiated ART was higher in our study (98.7%) than figures reported by the Chinese Centers for Disease Control and Prevention (92.9% among people living with HIV [35,36]) in 2020. This discrepancy may have been due to the difference in the period under observation. A previous study conducted in Sichuan, China, found that 30-day ART initiation declined early in the COVID-19 pandemic, but increased from 2021 to 2022 (i.e., when our study was conducted) [37]. Additionally, the downtown satellite of the SPHCC where we recruited study participants, was located near a metro station in Hongkou District, a central area of Shanghai, and provided a broad range of HIV services (ART, CD4 testing, viral load testing) and other specialist services. Furthermore, SCDC mobilized staff to contact people living with HIV with whom they had not regularly followed up, in accordance with national guidelines during the COVID-19 pandemic [38]. Together, these incentives and follow-up reminders may have helped to increase rates of ART initiation when compared with rate reported by the Chinese CDC.

The proportion of participants who were virally suppressed in our study was lower compared to other Chinese reports (72.8% in our full sample or 81.5% in our sensitivity analysis versus 96.1% [35]). This discrepancy may have been due to different definitions of viral suppression and the inclusion of all categories of internal migrants. Specifically, our study used a more stringent definition, defining viral suppression as less than 40 copies/mL, consistent with standards used at the clinic. Chinese guidelines and other reports, however, often used cutoffs of 400 copies/mL [39]. Our more stringent definition of viral suppression would have underestimated proportions attaining viral suppression compared with other reports. Additionally, our study likely included a higher proportion of internal migrants, particularly migrants with no residency status, compared with other reports of broader Chinese population. Compared with an average Chinese citizen, internal migrants experience high mobility [40] and HIV-related stigma [41] which may create barriers to care. Moreover, 16 participants did not undergo viral load testing and were conservatively classified as having a detectable/unknown viral load in our primary analysis.

In our study, the group of external permanent residents had the highest rate of viral suppression (85.7%) compared to 63.9-70.9% in the other residency status categories. In adjusted analyses, Shanghai Hukou residents, temporary migrants, and those without Shanghai residence identification were less likely to be virally suppressed compared to external permanent residents, but this association was no longer statistically significant after excluding the 16 participants without viral load test results from our analyses. Our findings align with changes in HIV care policies at the onset of the COVID-19 pandemic and findings from other studies. Prior to the COVID-19 pandemic, external permanent and Shanghai Hukou residents had free access to sexual health services such as HIV testing, care, and treatment whereas temporary migrants and those without Shanghai residency status had access to voluntary counselling and testing in Shanghai but were restricted in their access to HIV care and treatment in Shanghai and referred to their home province to access these services [24]. The onset of the COVID-19 pandemic prompted the policy change that allowed one month of free ART to anyone at any local HIV clinic or hospital regardless of residency status. Additionally, the new policy directed local clinics to contact clients for follow-up HIV care (e.g., ART and medication management) in accordance with national guidelines during the COVID-19 pandemic. This approach may have improved access to ART and, in doing so, increased the proportion of individuals who were able to attain viral suppression. Data have shown that ART coverage in China increased from 89.7% in 2019 to 92.6% in 2021, following these policy changes [38]. Similar proportions who were virally suppressed among Shanghai Hukou holders, temporary migrants, and men without Shanghai residence identification suggested that the new policy may have improved equitable access to HIV care, including access to ART. However, the lower odds of viral suppression among all groups compared to external permanent migrants suggest that inequities persisted. Specifically, external permanent residents were more likely than Hukou residents to be virally suppressed. This may be explained by the “healthy immigrant theory” which suggests that recent immigrants often have better health outcomes than native-born populations, despite facing additional challenges [42]. Further research designed to understand specific reasons for these differences among internal migrants would help to better understand this theory and inform future policies and programs to improve care for migrants and those with Hukou. Replication studies focused on residency status in other urban settings would also help to understand these broader dynamics regarding residency status.

In addition to the changes in HIV care policies upon the onset of the COVID-19 pandemic, most men without Shanghai residence identification in our study were involved in concurrent partnerships (72.7%), but self-identified as single/never married (85.5%). Being single was shown to be associated with reduced social support [26,43] which may lead to decreased access to HIV care and more difficulty attaining viral suppression. It has also been hypothesized that some married men initiate ART due to a sense of responsibility for protecting their health and the health of their primary partner [26]. By contrast, those who self-identified as single may not have felt the need to initiate ART to protect their primary sex partners. In our study, men without Shanghai residence identification were more commonly involved in concurrent partnerships despite lower odds of viral suppression, which suggests a potential risk of wider HIV transmission. Expansion of access to HIV care and health insurance while addressing key social determinants of health including residency status and social support are needed to support HIV prevention in Shanghai, especially among men without residency status [44]. Targeted messaging strategies are needed to ensure these men are informed of opportunities to access treatment and care.

Our study found that those who had a higher baseline CD4 count and fewer weeks from their last viral load test to the end of the study observation period were more likely to be virally suppressed. Consistent with existing evidence, baseline CD4 counts have often been used to distinguish early versus late HIV diagnoses [45]. In 2015, a recommendation for ART initiation immediately following an HIV diagnosis gained broad acceptance [46,47]. Following these guidelines, early HIV diagnosis would lead to earlier ART initiation, which was associated with durable viral suppression [48], improved health outcomes (e.g., decreased risk of severe disease and mortality [49,50]), and opportunities to prevent onward HIV transmission [51,52]. Furthermore, regular viral load testing was found to be a cost-effective method of identifying treatment failure prior to immunologic decline and to improve chances for treatment success [53]. Viral suppression was also associated with fewer healthcare visits [54], leading to reductions in healthcare costs. Our findings suggest a need to scale up of equitable policies and programs for early HIV diagnosis, early ART initiation, and viral load testing that prove to be effective for preventing onward HIV transmission and healthcare costs.

Our study had several limitations. The study setting was a single premier HIV treatment center in Shanghai, situated in a metropolitan setting, and focused on male internal migrants who could speak and understand Mandarin. These particulars may limit the generalizability of our findings as clients may have had better access to information and more awareness of HIV treatment and care. Participants’ HIV knowledge, including ART awareness and beliefs may have evolved over time, but this would not have been captured given the cross-sectional design. Rather than randomly sampling from a client list, we recruited clients who visited the downtown satellite of the SPHCC on days and times where a trained interviewer was available to administer the survey to sequential clients as they arrived at the clinic. Therefore, selection bias may have further limited the representativeness of our sample. In Shanghai, clients who initiated ART following an HIV diagnosis had access to free viral load and CD4 testing whereas those who did not initiate ART experienced barriers and variable access to these services. Given the high proportion of participants who initiated ART in our sample, our study may have overestimated the proportion of migrant men in Shanghai who access viral load testing. Additionally, there were variations in the implementation of public health restrictions and HIV care policy changes between jurisdictions during the COVID-19 pandemic which may further limit the generalizability of our findings to other regions in China. Shanghai residency status was based on self-report. Despite the fact that participants completed the survey with a trained interviewer, social desirability bias could have led to over-reporting of formal residency statuses [55,56]. This classification bias may have skewed findings towards the null effect, resulting in a more conservative estimate of the association between having no formal residency status and viral suppression. Despite follow-up phone calls to obtain viral load results, some participants were lost to follow-up, resulting in missing viral load and viral suppression data for 16 individuals. Finally, small sample size increased the risk of random error and reduced the precision of effect estimates leading to wider confidence intervals. Future studies should aim to examine associations between residency status, baseline CD4 count, missed opportunities for viral load testing, and undetectable viral load attainment among larger, more representative samples across a wider range of urban settings.

## Conclusions

As the Chinese government weighs the continuing provision of free HIV services to all citizens regardless of location and residency status, this study suggested that, while removing restrictions may have mitigated disparities, access to free ART alone did not eliminate differences in viral suppression attainment between residency groups. We had hypothesized that internal migrants without any residency status would be about as likely as regular Hukou residents to attain viral suppression during the period of altered service access policies during the COVID-19 pandemic. While the evidence supported this hypothesis, we found that external permanent migrants were more likely to attain viral suppression compared with other residency groups including Hukou residents. These findings suggest that more work is needed to understand the effects of residency status with the aim of designing interventions to improve viral suppression among migrants and Hukou residents alike. While inclusive ART policies can improve equitable access to treatment and testing, targeted messaging and other complementary interventions that take account of the unique challenges faced by all people regardless of residency status are needed to ensure optimal public health impact. Further research will support this aim by examining the barriers linked to residency status that may hinder rapid attainment of viral suppression across urban settings in China and elsewhere.

## Supporting information

S1 File S1 checklist. (DOCX)
